# Visual quality of juvenile myopes wearing multifocal soft contact lenses

**DOI:** 10.1186/s40662-020-00204-4

**Published:** 2020-07-19

**Authors:** Xiaopeng Huang, Feifu Wang, Zhiyi Lin, Yifan He, Shuyun Wen, Ling Zhou, Fan Lu, Jun Jiang

**Affiliations:** School of Ophthalmology and Optometry, Wenzhou Medical University, 270 Xueyuan West Road, Wenzhou, 325027 Zhejiang People’s Republic of China

**Keywords:** Myopia, Visual quality, Multifocal soft contact lenses (MFSCLs), Contrast visual acuity, Aberrations

## Abstract

**Background:**

It is unclear whether multifocal soft contact lenses (MFSCLs) affect visual quality when they are used for myopia control in juvenile myopes. The aim of this study was, therefore, to investigate the effect of MFSCLs on visual quality among juvenile myopia subjects.

**Methods:**

In a prospective, intervention study, thirty-three juvenile myopes were enrolled. Visual perception was assessed by a quality of vision (QoV) questionnaire with spectacles at baseline and after 1 month of MFSCL wear. At the one-month visit, the high (96%) contrast distance visual acuity (distance HCVA) and low (10%) contrast distance visual acuity (distance LCVA) were measured with single vision spectacle lenses, single vision soft contact lenses (SVSCLs) and MFSCLs in a random order. Wavefront aberrations were measured with SVSCLs, with MFSCLs, and without any correction.

**Results:**

Neither distance HCVA (*p* > 0.05) nor distance LCVA (*p* > 0.05) revealed any significant difference between MFSCLs, SVSCLs and single vision spectacle lenses. The overall score (the sum of ten symptoms) of the QoV questionnaire did not show a statistically significant difference between spectacles at baseline and after 1 month of MFSCL wear (*p* = 0.357). The results showed that the frequency (*p* < 0.001), severity (*p* = 0.001) and bothersome degree (*p* = 0.016) of halos were significantly worse when wearing MFSCLs than when wearing single vision spectacle lenses. In contrast, the bothersome degree caused by focusing difficulty (*p* = 0.046) and the frequency of difficulty in judging distance or depth perception (*p* = 0.046) were better when wearing MFSCLs than when wearing single vision spectacle lenses. Compared with the naked eye, MFSCLs increased the total aberrations (*p* < 0.001), higher-order aberrations (*p* < 0.001), trefoil (*p* = 0.023), coma aberrations (*p* < 0.001) and spherical aberrations (SA) (*p* < 0.001). Compared with the SVSCLs, MFSCLs increased the total aberrations (*p* < 0.001), higher-order aberrations (*p* < 0.001), coma aberrations (*p* < 0.001) and SA (*p* < 0.001). The direction of SA was more positive (*p* < 0.001) with the MFSCLs and more negative (*p* = 0.001) with the SVSCLs compared with the naked eye.

**Conclusions:**

Wearing MFSCLs can provide satisfactory corrected visual acuity (both distance HCVA and distance LCVA). Although the lenses increased the aberrations, such as total aberrations and higher-order aberrations, there were few adverse effects on the distance HCVA, distance LCVA and visual perception after 1 month of MFSCL use.

**Trial registration:**

Chinese Clinical Trial Registry: ChiCTR-OOC-17012103. Registered 23 July 2017, http://www.chictr.org.cn/usercenter.aspx

## Background

In the past few decades, the prevalence of myopia has increased rapidly worldwide, especially in Asian countries, where it has risen even to 80–90% [1–3]. More concerning is that myopia tends to occur in younger individuals, and the proportion of high myopic cases is increasing [4–6]. High myopia is associated with some ocular diseases causing blindness, such as cataracts, glaucoma, macular degeneration, retinal detachment, and choroidal neovascularization [7–9]. Myopia control has been a hotspot worldwide, especially in East Asia. To date, various methods have been used for myopia control, such as orthokeratology [10, 11], soft contact lenses [12, 13] and low-dose atropine [14–17].

Some studies have shown that multifocal soft contact lenses (MFSCLs) have a positive effect on myopia control [18–20]. The studies by Pauné [18] and Walline [19] concluded that MFSCLs provided a better therapeutic effect than single vision spectacle lenses by follow-up after 2 years. A 3-year randomized clinical trial demonstrated that MiSight soft contact lenses with dual-focus optics were effective in slowing the progression of myopia in enrolled subjects from 8 to 12 years old [20].

The mechanism by which myopia progression is slowed is unclear. One theory supported by many researchers is that peripheral myopic defocus slows the progression of myopia [21–23], which was confirmed by Benavente-Perez’s research in marmosets [24].

MFSCLs have different power profiles in the optical zone [25]. Generally, there are two center distance designs of contact lenses that are designed to slow the progression of myopia: concentric ring design of bifocal contact lenses and progressive design of MFSCLs [26].

MFSCLs are used for correction of presbyopia [27, 28] to obtain good vision at all distances. However, some studies [29] suggested that wearing MFSCLs may lead to adverse effects on visual quality and disturbing visual symptoms such as halo and glare, especially at night when the pupils are larger.

It is unclear whether MFSCLs affect visual quality when MFSCLs are used to slow the progression of myopia in juvenile myopes patients. To date, few studies have been conducted on the effect on children’s visual quality after wearing MFSCLs for a long time.

The primary aim of this study was, therefore, to investigate the effect of MFSCLs on visual quality in juvenile myopia subjects by measuring high contrast distance visual acuity (distance HCVA, 96%), low contrast distance visual acuity (distance LCVA, 10%), and quality of vision (QoV) questionnaire for subjective visual performance as well as wavefront aberration assessment to objectively understand the impact on optical imaging quality.

## Methods

### Subjects

From July 1 to October 31, 2017, at the Eye Hospital of Wenzhou Medical University, thirty-three juvenile myopic patients were enrolled, 11 males and 22 females. Table 1 provides the inclusion and exclusion criteria.

**Table 1:** Inclusion and exclusion criteria

*Inclusion Criteria*	
1. Age from 8 to 14 years old.	
2. Spherical equivalent between −1.00 D and − 8.00 D.	
3. Astigmatism of no greater than 1.50 D.	
4. Myopic progression in the last year of greater than 0.50 D.	
5. Flat corneal curvature between 40.00 D and 46.00 D.	
*Exclusion Criteria*	
1. Pre-existing systemic disease that had an influence on contact lens wear.	
2. Ocular injury or surgery.	
3. Contraindication for MFSCL fitting.	
4. Have worn other contact lenses.	
5. Pupil in dark environment smaller than the minimum diameter required to measure aberrations for 6 mm.	

*MFSCL* = multifocal soft contact lens

### Study design

This was a prospective intervention study that adhered to the tenets of the Declaration of Helsinki. Ethics approval (2017–5-Q-4) was granted by the Ethics Committee of Eye Hospital, Wenzhou Medical University. A detailed explanation of all possible risks was provided to the subjects’ parents, and informed consent was obtained before the study.

Before MFSCLs were fitted, some measurements were completed, including subjective refraction, corneal topography and intraocular pressure. The anterior ocular surface was examined by slit-lamp biomicroscopy. The trial lens was selected based on the vertexed spherical equivalent (SE) of the subjective refraction and the lens fitting was evaluated by slit-lamp biomicroscopy. If fitting of the trial lens was not acceptable, the subject was excluded. The final prescription was determined after over-refraction. The subjects were required to wear the lens for at least 8 h a day for 5 days a week. During subsequent follow-up times, visual quality was evaluated from two aspects: subjective assessment (including distance HCVA, distance LCVA and the QoV questionnaire) and objective evaluation (wavefront aberration assessment).

The difference in the effect on visual perception was studied by comparing the scores of the QoV questionnaire between MFSCLs and new backup single-vision spectacle lenses. All subjects completed the QoV questionnaire twice. The first time was at baseline to evaluate the visual perception with new backup spectacles. The prescriptions of new spectacles were based on subjective refraction and the questionnaire was completed before MFSCL fitting. The second time was at the one-month follow-up to evaluate the visual perception with MFSCLs. In addition, an intervention study was carried out to analyze other indicators. The distance HCVA and distance LCVA, corrected by single-vision spectacle lenses, SVSCLs and MFSCLs, were measured to analyze the effect on visual quality on the same day after 1 month of wearing MFSCLs. It was also necessary to measure wavefront aberrations with SVSCLs and MFSCLs, as well as without any correction, to analyze visual quality objectively. Since the subjects were minors, they were required to wear and care for the lenses with the help of their guardians.

### Lenses

#### MFSCLs

The MFSCLs used in this study were BioThin (Taiwan, China), which are daily disposable contact lenses. These lenses were composed of Ocufilcon D material and had a base curve of 8.6 mm, water content of 55%, lens diameter of 14.2 mm, center thickness of 0.08 mm, DK of 19 × 10^− 11^ (cm^2^/s) (mlO_2_/ml × mmHg), and refractive index of 1.409.

Compared with SVSCLs, MFSCLs are designed specifically to provide adequate refractive power to correct myopia in the central optical zone approximately 3 mm in diameter and provide relatively positive power to produce myopic defocus in the peripheral optical zone approximately 6 mm in diameter [30].

**Fig. 1:**
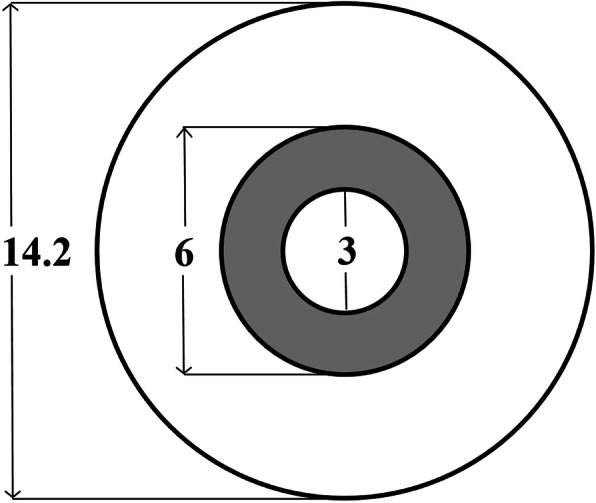
Design of multifocal soft contact lenses (mm)

**Fig. 2:**
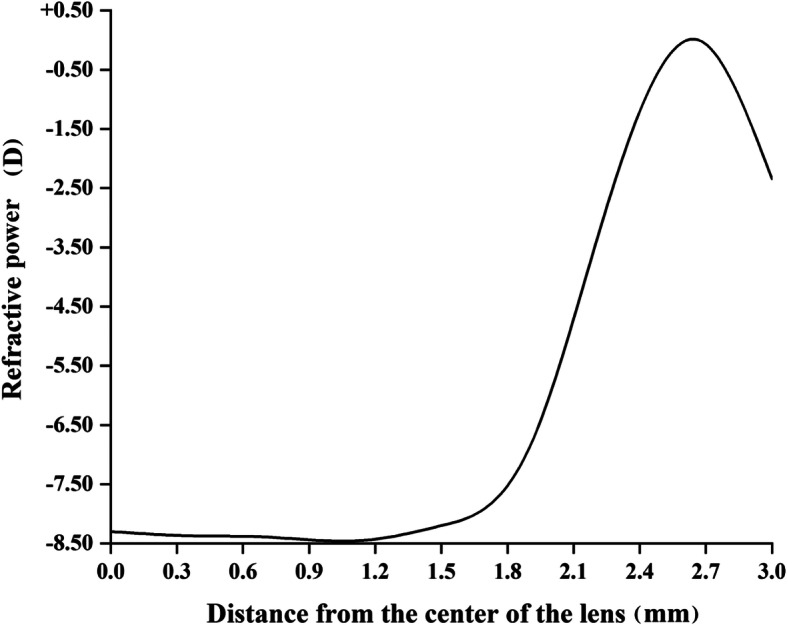
Radial power profile (D) of multifocal soft contact lenses

Figure 1 shows a sketch of the spherical design of the central correction area and the aspherical continuous progressive design of the lens control area. Figure 2 shows a sample graph of MFSCLs with − 8.50D distance power to show the radial power profile within a central 3-mm radius.

#### Single vision soft contact lenses (SVSCLs)

The SVSCLs used in this study were ACUVUE (Johnson & Johnson, State of New Jersey, USA), the daily disposable contact lenses.

These lenses were composed of Etafilcon A material and had an 8.5-mm base curve. These had water content of 58%, lens diameter of 14.2 mm, center thickness of 0.084 mm, DK of 28 × 10^− 11^ (cm^2^/s) (mlO_2_/ml × mmHg), and refractive index of 1.400.

#### Single vision spectacle lenses

The spectacles used in this study were glasses with a refractive index of 1.523, determined by subjective refraction. All subjects received new spectacles on the day of baseline.

### Measurements

#### Diopter

The subjective refraction was measured by a phoropter (TOPCON, Japan).

#### Distance HCVA and distance LCVA

LogMAR visual acuity was assessed under high (96%)/low (10%) contrast at a distance (4 m) by a Landolt C device (Precision Vision, USA). A photometer (SEKONIC L-758, Japan) was used to measure the illumination intensity to obtain a brightness of the symbol of 10.2–10.5 EV and an ambient luminance of 7.7 to 8.1 EV before each visual measurement.

The distance HCVA and LCVA of the right eye was measured with single vision spectacle lenses, SVSCLs, and MFSCLs. The participants wore MFSCLs daily for 1 month. They wore each type of correction at the one-month visit for at least 10 to 15 min in a random order, prior to measurements being taken. Randomization was completed by numerical randomization. There was an interval of at least 10 min (no lens over eyes) from wearing one type of lens to another to minimize the effects of prior correction. The participants were required to distinguish the smaller symbols until half of the line was incorrectly distinguished. We recorded the results by LogMAR visual acuity (0.02 LogMAR per symbol was distinguished).

#### QoV questionnaire

The participants’ visual perception was evaluated by McAlinden’s visual quality scale (QoV), which was translated to Chinese by Jinhai Huang [31]. This scale was used to verify the validity, accuracy and reliability of visual quality evaluation by statistical methods, including that of Rasch.

The questionnaire was completed with spectacles at baseline and MFSCLs at the one-month follow-up visit. There were ten symptoms, including glare, halos, starbursts, hazy vision, blurred vision, distortion, double vision, visual fluctuation, focusing difficulty, and difficulty in judging distance or depth perception. Each item contains three subscales, namely, frequency, severity, and bothersome degree. For the first seven symptoms, an accompanying image was developed to aid in understanding the questions and to reduce the possibility of inconsistent responses. Each question had four options (A, B, C, D) with descriptive words, including never (A), occasionally (B), quite often (C), very often (D) for frequency; not at all (A), mild (B), moderate (C), severe (D) for severity; and not at all (A), a little (B), quite (C), very (D) for bothersome degree. They corresponded to scale values of 0, 1, 2 and 3, respectively. The total scores of each subject were summed; the higher the score, the worse the visual quality [31].

#### Wavefront aberrations

The aberrations of the right eye were measured in dim illumination by a Shack-Hartmann wavefront aberrations instrument (WASCA, Carl Zeiss Meditec, Germany). The participants daily wore MFSCLs for 1 month. They wore each type of correction (SVSCLs, MFSCLs, and without any correction) for at least 10 to 15 min, prior to measurements being taken. The order of wearing SVSCLs, MFSCLs, and no correction was random. Randomization was completed by numerical randomization. There was an interval time of at least 10 min (no lens over eyes) from one type of lens to another.

The aim of this test was to calculate the full-eye aberrations by the difference between the ideal wavefront aberrations and the actual plane image.

When calculating the root mean square (RMS) value of wavefront aberrations, we stripped out the first-order aberrations, which had no effect on image quality, and the second-order spherical defocusing, which could be corrected by a spherical contact lens.

When measured, the absolute deviations of the pupil center in the x-axis and y-axis should be less than 0.4 mm. Aberrations of a 7-order 35-term Zernike polynomial were measured within a pupillary area of 6 mm in diameter. The measurement of aberrations was repeated three times and we averaged the three measurements. The average RMS value of the wavefront aberrations were used as an indication to estimate the deviation between the ideal and actual images. The higher the RMS value, the poorer the retinal imaging quality.

### Statistical analysis

Statistical analysis was performed using SPSS 22 (New York, NY). The normality analysis of data was based on the Kolmogorov-Smirnov (K-S) test. The difference between different ways of correction was compared by repeated measures ANOVA. The Wilcoxon signed rank test was used to analyze the difference in the QoV questionnaire between spectacles at baseline and MFSCLs at the one-month follow-up visit.

## Results

### Baseline data

Thirty-three juvenile myopes were enrolled, 11 males and 22 females, with age of 11.30 ± 1.50 years, SE of − 2.59 ± 1.07 D, astigmatism of 0.20 ± 0.25 D, and corneal toricity of 1.14 ± 0.48 D.

### Distance HCVA and distance LCVA

The distance HCVA and LCVA of the right eye were measured in three corrected ways: single vision spectacle lenses, SVSCLs and MFSCLs. Analyzed by repeated measures ANOVA (Bonferroni test), the difference between these three methods did not reach statistical significance for either HCVA (*p* > 0.05) or LCVA (*p* > 0.05). Table 2 shows HCVA and LCVA of three myopia correction methods.

**Table 2:** High and low contrast visual acuity (mean ± SD) of three myopia correction methods

Lenses	N	Distance HCVA (LogMAR)	Distance LCVA (LogMAR)
Single vision spectacle lenses	33	0.01 ± 0.11	0.24 ± 0.12
SVSCLs	33	0.02 ± 0.15	0.23 ± 0.16
MFSCLs	33	−0.04 ± 0.10	0.19 ± 0.10

*Distance HCVA* = high contrast distance visual acuity, *Distance LCVA* = low contrast distance visual acuity, *SVSCLs* = single vision soft contact lenses, *MFSCLs* = multifocal soft contact lenses

### QoV questionnaire

The higher the score, the worse the visual quality. This study compared the scores of the QoV questionnaire between wearing new backup single vision spectacle lenses at baseline and wearing MFSCLs after one-month of wear. The Wilcoxon signed rank test was used for the analysis. The results showed that the frequency (0.36 ± 0.60 spectacles vs. 1.3 ± 1.13 MFSCLs, *p* < 0.001), severity (0.27 ± 0.52 spectacles vs. 0.94 ± 0.90 MFSCLs, *p* = 0.001) and bothersome degree (0.15 ± 0.44 spectacles vs. 0.61 ± 0.83 MFSCLs, *p* = 0.016) of halos were significantly worse when wearing MFSCLs than when wearing single vision spectacle lenses. In contrast, the bothersome degree caused by focusing difficulty (0.15 ± 0.36 spectacles vs. 0.03 ± 0.17 MFSCLs, *p* = 0.046) and the frequency of difficulty in judging distance or depth perception (0.15 ± 0.36 spectacles vs. 0.03 ± 0.17 MFSCLs, *p* = 0.046) were better when wearing MFSCLs than when wearing single vision spectacle lenses. Other symptoms, including glare, starbursts, hazy vision, blurred vision, distortion, double vision, and visual fluctuation, did not show a statistically significant difference (*p* > 0.05).

In summary, the overall score (the sum of ten symptoms, ranging from 6.97 ± 5.67 to 7.42 ± 5.95) of the QoV questionnaire did not show a statistically significant difference between spectacles at baseline and after one-month of MFSCL wear (*p* = 0.357).

### Wavefront aberrations

**Fig. 3:**
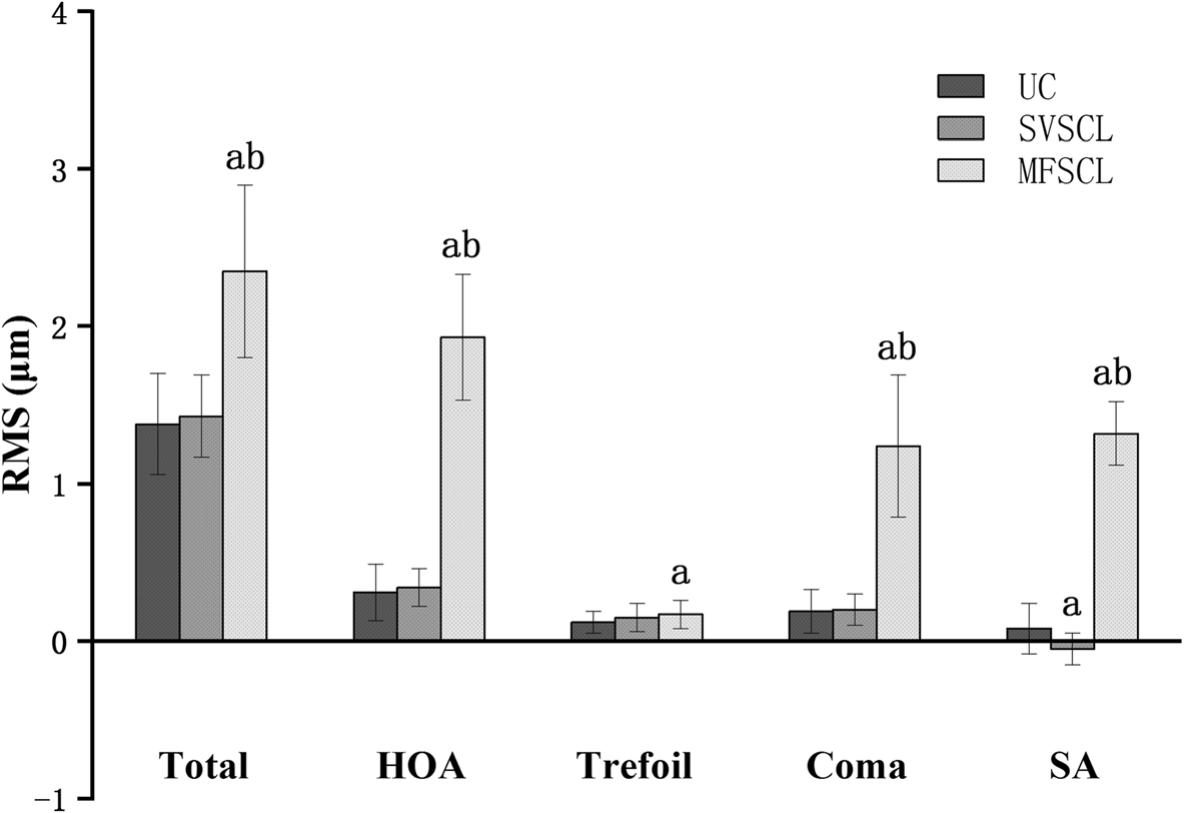
Aberrations under different correction methods. RMS: root mean square; UC: uncorrected (naked eyes); Total: total aberrations (2nd-to-7th-order aberrations, removing second-order defocus); HOA: higher-order aberrations (including 3rd-to-7th-order aberrations); Trefoil: trefoil aberrations (including C (3, − 3) and C (3, 3)); Coma: coma aberrations (including C (3, − 1) and C (3, 1)); SA: spherical aberrations (C (4, 0)). “a” indicates statistical significance (*p* < 0.05) compared with the naked eye, and “b” indicates statistical significance (*p* < 0.05) compared with single vision soft contact lenses (SVSCLs)

The RMS values of the aberrations were compared using repeated measures ANOVA and corrected with Bonferroni. Compared with the naked eye, aberrations of MFSCLs were significantly increased in total aberrations (*p* < 0.001), higher-order aberrations (*p* < 0.001), trefoil (*p* = 0.023), coma (*p* < 0.001) and spherical aberrations (SA) (*p* < 0.001). Compared with the SVSCLs, aberrations of MFSCLs were significantly increased in total aberrations (*p* < 0.001), higher-order aberrations (*p* < 0.001), coma (*p* < 0.001) and SA (*p* < 0.001). In addition, there was more SA with the naked eye than with SVSCLs (*p* = 0.001). The direction of SA was more negative with SVSCLs (mean SA = − 0.05 ± 0.10 μm) than the naked eye (mean SA = 0.08 ± 0.16 μm), whereas that of the MFSCLs was more positive (mean SA = 1.32 ± 0.20 μm). Figure 3 shows the aberrations under different correction methods.

## Discussion

In this report, the visual quality after wearing MFSCLs was evaluated from two aspects: subjective assessment (including distance HCVA, distance LCVA and the QoV questionnaire), and objective evaluation (wavefront aberration assessment).

### Distance HCVA and distance LCVA

Here, we demonstrate that neither HCVA nor LCVA were significantly different between MFSCLs, SVSCLs and spectacles at the one-month follow-up visit, suggesting that MFSCLs can provide similar distance visual acuity to that of SVSCLs and spectacles. The studies by Kollbaum et al. [32] and Kang et al. [33] showed that there was no significant difference in HCVA between MFSCLs and spectacles, which was similar to our HCVA results.

However, Kollbaum’s study showed that distance LCVA with MFSCLs was worse with spectacles at baseline, and Kang’s study showed that distance LCVA with MFSCLs was worse with spectacles after 2 weeks of MFSCL wear. These differences could be attributed to two factors: First, in our study, subjects had a relatively longer time for adaption to MFSCLs than previous studies as we evaluated distance LCVA at the 1-month visit. We presumed that subjects had adapted to the distance visual acuity after daily wearing of MFSCLs for 1 month, which may affect distance visual acuity. Second, the result may be related to lens design. The central optical zone of the lenses used in our study was 3.0 mm, while that in their studies (Kollbaum et al. [32] and Kang et al. [33]) was 2.3 mm. The MFSCLs, with a larger diameter of the central optical zone, may achieve better corrected distance visual acuity. Therefore, we assumed that subjects could achieve similar distance HCVA and LCVA with MFSCLs as well as SVSCLs and spectacles after a long adaptation and large central optic zone of MFSCLs.

### QoV questionnaire

There was no difference in the total score of the QoV questionnaire between wearing MFSCLs and spectacles, indicating that MFSCLs may provide a similar visual perception to that of spectacles, which is inconsistent with previous studies. Others [32, 34, 35] showed that MFSCLs had negative effects on visual perception compared with spectacles. When wearing MFSCLs, visual acuity deteriorates and visual symptoms, such as glares, become more serious in the early stage. Kang’s study [33] compared the visual perception of MFSCLs and SVSCLs by the same QoV questionnaire as that used in the present study. Their study showed that MFSCLs yielded poorer visual quality than SVSCLs based on the results of the QoV questionnaire. The studies mentioned above were all short-term studies from 1 h to 2 weeks. In our study, all subjects completed the QoV questionnaire for MFSCLs at the one-month follow-up visit. Therefore, we assumed that with a longer adaption to MFSCLs, visual perception may improve. In addition, studies have shown the prolongation of exposure to visual interference, and these adverse reactions may improve depending on fuzzy adaptation by the brain with increased focal depth [36–38].

To specify which visual symptoms were affected most with MFSCLs, we analyzed the frequency, severity and disturbance of each symptom in the present study. Our results showed that compared with wearing spectacles at baseline, there was more evidence of halos with MFSCLs after 1 month of daily wear of MFSCLs. It may be explained by the design of the MFSCLs. The MFSCL has an aspherical continuous progressive design in the peripheral optical zone. Thus, if subjects have large pupils, they may experience more myopic defocus and halos, especially under dim illumination [29]. All subjects were adolescents who had relatively large pupils, which may also make them susceptible to halos. Meanwhile, compared with spectacles, some symptoms improved when wearing MFSCLs. The bothersome degree caused by focusing difficulty was better when wearing MFSCLs than spectacles. Additionally, when wearing MFSCLs, the frequency of difficulty in judging distance or depth perception was better than with spectacles. Compared with spectacles, MFSCLs could provide a more realistic size of objects. Furthermore, these lenses could follow the eyes in all directions when the eyes rolled.

### Wavefront aberrations

Here, the astigmatism based on subjective refraction of the subjects was very mild, with a mean value of − 0.20 ± 0.25 D. The decentration of lenses was found in only one subject. During the one-week and one-month follow-up, the lens in one patient showed slight decentration, which was acceptable. The lens position was central in all the other subjects.

The results in this study showed that SVSCLs had little effect on aberrations, compared with the naked eye. Compared with SVSCLs, MFSCLs increased the total aberrations and higher-order aberrations, especially SA and coma aberrations.

SA was positive when wearing MFSCLs, while SA was negative when wearing SVSCLs, which is consistent with previous reports. Fedtke’s study [35] found that the center-distance MFSCLs induced positive SA. It is well known that MFSCLs provide peripheral myopic defocus based on their peripheral aspheric continuous progressive design. The positive SA may be related to the unique design of MFSCLs [39].

Coma aberrations may be related to the lens movement and rotation [40]. Compared with the spherical design of SVSCLs, the aspheric continuous progressive design of MFSCLs made a greater impact on coma aberrations. A previous study by Fedtke et al. [34] also supported the finding that coma aberrations were more serious in MFSCLs than SVSCLs.

Ruru Chen et al. [41] recently showed that the decentration of the OrthoK treatment zone has a slightly positive effect on myopia control. This may be related to the aberrations caused by the decentration of orthokeratology. Similarly, this may also be the case with MFSCLs as the study MFSCLs were designed to produce a similar peripheral defocus in the front surface. In the future, more scientific material and design of lenses should be found to balance myopia control and visual quality. Reducing the negative impact on visual quality as well as ensuring the positive effect on myopia control, could enable MFSCLs to play a greater role in the clinic.

## Conclusions

MFSCLs were initially used to correct presbyopia and they are now also used for myopia control in children. This study investigated the effect of MFSCLs on the visual quality of children. The results demonstrated that wearing MFSCLs can provide satisfactory corrected visual acuity (both distance HCVA and distance LCVA). MFSCLs increased the aberrations, such as total aberrations and higher-order aberrations, but there were few adverse effects on distance HCVA, distance LCVA and visual perception after 1 month of wear of MFSCLs. MFSCLs are acceptable to children for myopia control because they can provide good visual quality.

## Data Availability

All data generated or analyzed during this study are included in this published article.
